# Supplementary material for: The SARS-CoV-2 Delta variant induces an antibody response largely focused on class 1 and 2 antibody epitopes
Source: bioRxiv. 2022 Mar 14:2022.03.12.484088. Preprint. [Version 1] doi: 10.1101/2022.03.12.484088 (PMC8936118; doi:10.1101/2022.03.12.484088)
Supplement: 1 [file NIHPP2022.03.12.484088V1-supplement-1.pdf]

bioRxiv preprint doi: https://doi.org/10.1101/2022.03.12.484088; this version posted March 14, 2022. The copyright holder for this preprint (which was not certified by peer review) is the author/funder, who has granted bioRxiv a license to display the preprint in perpetuity. It is made available under aCC-BY 4.0 International license.

Supplementary Information

S1 Table. Summary characteristics of cohorts examined in this study

Exposure history	Time period of sample collection	Location	Days post-symptom onset:	Number of individuals	Age	Number of females (%)	Study in which results first described	Types of data examined
Primary Delta infection	August–September, 2021	South Africa	mean 33.4 (range 24–37)	8	Mean 47.4 (range 36–57)	3/8 (37.5%)	Present study	Neutralization, DMS antibody-escape mapping
Delta breakthrough infection after 2x mRNA vaccination	July–September, 2021	Washington State, USA	Mean 28.3 (range 24–38)	8	Mean 29.6 (range 20–68)	5/8 (62.5%)	Present study	Neutralization, DMS antibody-escape mapping
Primary Beta infection	December 2020–January 2021	South Africa	Mean 32.9 (range 27–40)	9	Mean 54.1 (range 26–78)	5/9 (55.5%)	(12)	Neutralization, DMS antibody-escape mapping
Early 2020 infection	Prior to March 15, 2020	Washington State, USA	Mean 31.6 (range 15–61)	17	Mean 51.6 (range 23–76)	8/17 (47.1%)	(23)	Neutralization, DMS antibody-escape mapping
Early 2020 infection	Early 2020	New York, USA (65)	Mean 29 (range 21–35)	5	Mean 51.6 (range 42–66)	2/5 (40%)	(24)	DMS antibody-escape mapping
2x mRNA-1273*	Mid-2020	USA (mRNA-1273 phase I trial (66))	30 days post-dose 1	14 (after exclusion of one)*	Mean 31 (range recruited: 18–55)	9/15 (60%)	(29)	Neutralization, DMS antibody-escape mapping
2x BNT162b2	Early 2021	Washington State, USA	Mean 31.2 days post-dose 1 (range 28–34)	8	Mean 34.5 (range 28–50)	5/8 (62.5%)	Present study	Neutralization, DMS antibody-escape mapping

*One participant was excluded from analysis due to not receiving a second vaccination dose but their demographic information is unknown.

bioRxiv preprint doi: https://doi.org/10.1101/2022.03.12.484088; this version posted March 14, 2022. The copyright holder for this preprint (which was not certified by peer review) is the author/funder, who has granted bioRxiv a license to display the preprint in perpetuity. It is made available under aCC-BY 4.0 International license.

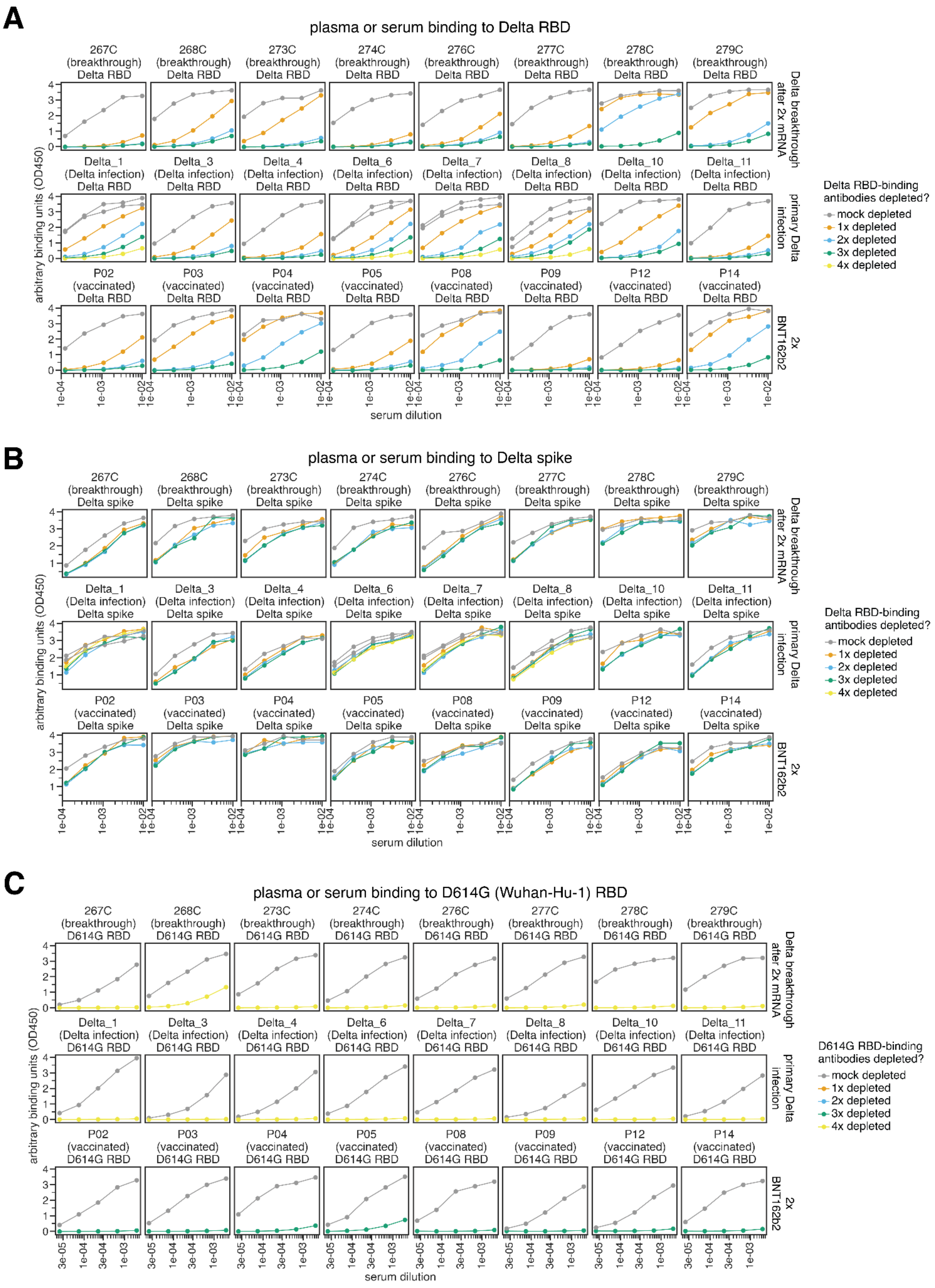

bioRxiv preprint doi: https://doi.org/10.1101/2022.03.12.484088; this version posted March 14, 2022. The copyright holder for this preprint (which was not certified by peer review) is the author/funder, who has granted bioRxiv a license to display the preprint in perpetuity. It is made available under aCC-BY 4.0 International license.

S1 Fig. Enzyme-linked immunosorbent assay (ELISA) of plasmas or sera before and after depletion of Wuhan-Hu-1 or Delta RBD-binding antibodies. Plasma or serum binding to the **(A)** Delta RBD or **(B)** Delta spike after mock or depletion of Delta RBD-binding antibodies. **(C)** Plasma or serum binding to D614G RBD after mock or depletion of D614G RBD-binding antibodies. Note that the D614G RBD is identical to the Wuhan-Hu-1 RBD.

bioRxiv preprint doi: https://doi.org/10.1101/2022.03.12.484088; this version posted March 14, 2022. The copyright holder for this preprint (which was not certified by peer review) is the author/funder, who has granted bioRxiv a license to display the preprint in perpetuity. It is made available under aCC-BY 4.0 International license.

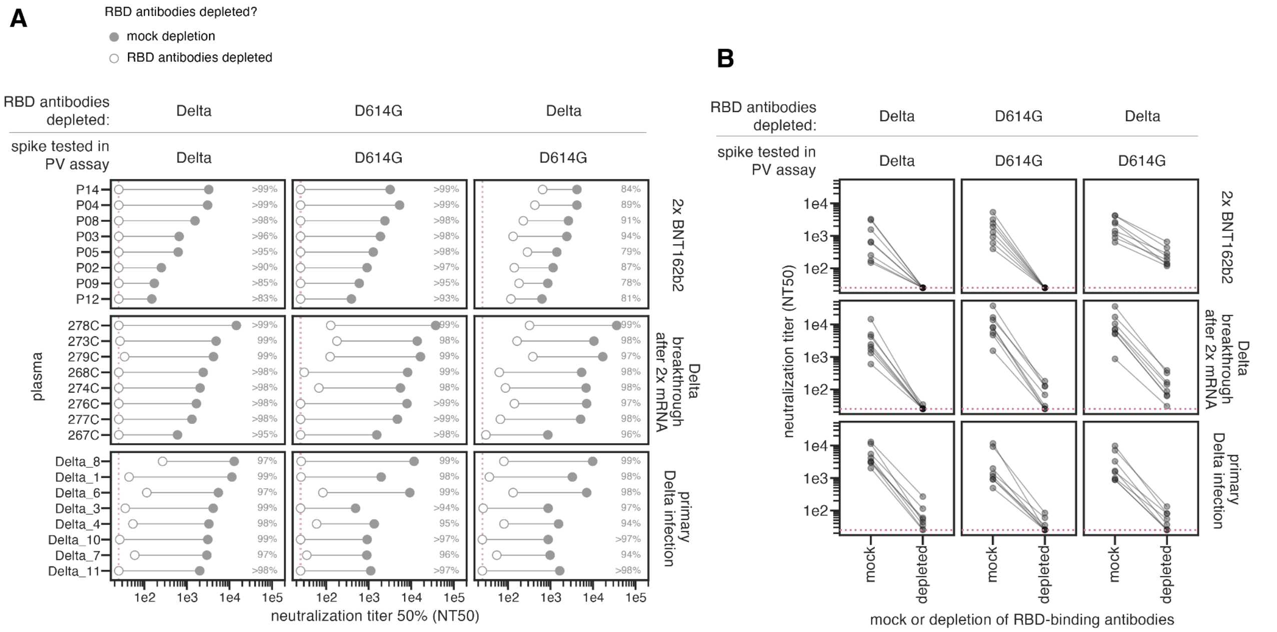

S2 Fig. Plasma neutralization of spike-pseudotyped lentiviral particles before and after depletion of Wuhan-Hu-1 (D614G) or Delta RBD-binding antibodies. Plasma neutralization titer 50% (NT50) of D614G or Delta spike-pseudotyped lentiviral particles for mock depletion or depletion of Wuhan-Hu-1 (D614G) or Delta RBD-binding antibodies. The labels above the plots indicate which RBD-binding antibodies were depleted and which spikes were pseudotyped on lentiviral particles. The same data are plotted by individual serum/plasma sample in **(A)** or grouped by cohort in **(B)**. A subset of these results are shown grouped by cohort in **Fig 2.** For plasmas depleted of Delta RBD-binding antibodies tested against D614G spike, neutralizing activity could be due to RBD-binding antibodies that do not bind the Delta RBD, or due to non-RBD-binding antibodies. The full neutralization curves are in **S7A Fig**. All neutralization data are in **S1 Data** and online at https://github.com/jbloomlab/SARS-CoV-2-RBD_Delta/blob/main/experimental_data/results/neut_titers/combined_neut_titers.csv.

bioRxiv preprint doi: https://doi.org/10.1101/2022.03.12.484088; this version posted March 14, 2022. The copyright holder for this preprint (which was not certified by peer review) is the author/funder, who has granted bioRxiv a license to display the preprint in perpetuity. It is made available under aCC-BY 4.0 International license.

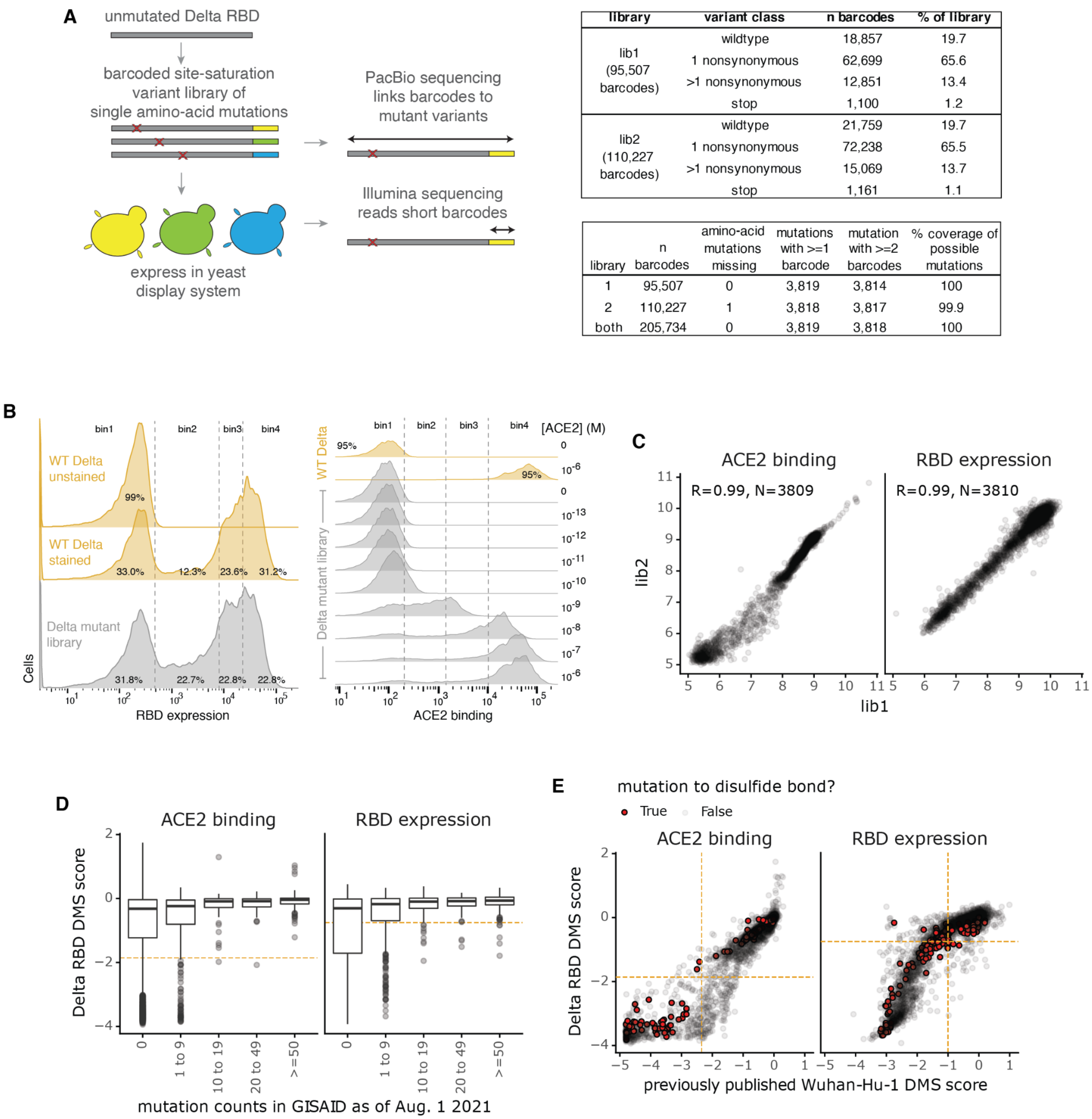

library	variant class	n barcodes	% of library
lib1 (95,507 barcodes)	wildtype	18,857	19.7
	1 nonsynonymous	62,699	65.6
	>1 nonsynonymous	12,851	13.4
	stop	1,100	1.2
lib2 (110,227 barcodes)	wildtype	21,759	19.7
	1 nonsynonymous	72,238	65.5
	>1 nonsynonymous	15,069	13.7
	stop	1,161	1.1

library	n barcodes	amino-acid mutations missing	mutations with >=1 barcode	mutation with >=2 barcodes	% coverage of possible mutations
1	95,507	0	3,819	3,814	100
2	110,227	1	3,818	3,817	99.9
both	205,734	0	3,819	3,818	100

S3 Fig. Generation of the Delta RBD mutant libraries and measurements of effects of mutations on ACE2 binding and RBD expression. (A) Schematic showing the Delta RBD mutant library design. A site-saturation variant library was generated in the Delta RBD background, targeting one amino-acid mutation per variant. Nx16 unique DNA barcodes were appended to the variant gene fragments. The Nx16 barcodes were linked to their associated RBD mutations by PacBio circular consensus sequencing (CCS). The plasmid library DNA was transformed into yeast cells. In downstream experiments, the Nx16 barcodes are sequenced by short-read Illumina sequencing. The tables at right indicate key library statistics. **(B)** FACS gating scheme used to measure effects of RBD mutations on expression on the yeast cell surface

bioRxiv preprint doi: https://doi.org/10.1101/2022.03.12.484088; this version posted March 14, 2022. The copyright holder for this preprint (which was not certified by peer review) is the author/funder, who has granted bioRxiv a license to display the preprint in perpetuity. It is made available under aCC-BY 4.0 International license.

(left) or on binding to monomeric ACE2 (right). The ACE2 binding and expression scores are in **S2 Data** and online at https://github.com/jbloomlab/SARS-CoV-2-RBD_Delta/blob/main/results/final_variant_scores/final_variant_scores.csv. These results are summarized in greater detail in (36). **(C)** Correlations between biological independent replicate library measurements of the effects of single mutations on ACE2 binding and RBD expression, measured as described in (53). See **Methods** for experimental details. **(D)** Thresholds on the ACE2 binding and RBD expression scores (dashed orange lines) for the Delta mutant library to computationally filter highly deleterious variants that may represent spurious antibody-escape mutations. We aimed to retain most mutations that have been observed >=50 times in sequenced SARS-CoV-2 isolates. The x-axis categorizes mutations by their number of observations in GISAID (67) as of Aug. 1, 2021. An ACE2 binding score threshold of >= –1.86 ($10^{-1.86}$ binding compared to unmutated Delta RBD, which is a 72.4-fold loss in binding affinity) and an RBD expression score of >= –0.75 ($10^{-0.75}$ expression compared to unmutated Delta RBD, which is a 5.6-fold loss in RBD expression) were chosen, which filter comparable numbers of mutations as in prior Wuhan-Hu-1 experiments (23,43). These filters retain 100 and 97.3% of mutations, respectively, that have been observed >=50 times in sequenced SARS-CoV-2 isolates. **(E)** Relationship between the ACE2 binding and RBD expression scores for the Delta RBD library compared to those previously published for the Wuhan-Hu-1 library in Starr, et al. (2020) (53). The computational filters used for antibody-escape experiments for the Wuhan-Hu-1 (23) and Delta libraries are dashed orange lines. Each dot is one mutation, and mutations to disulfide bonds are shown in red. A key difference is that for the previously published Wuhan-Hu-1 experiments, dimeric rather than monomeric ACE2 was used (53).

bioRxiv preprint doi: https://doi.org/10.1101/2022.03.12.484088; this version posted March 14, 2022. The copyright holder for this preprint (which was not certified by peer review) is the author/funder, who has granted bioRxiv a license to display the preprint in perpetuity. It is made available under aCC-BY 4.0 International license.

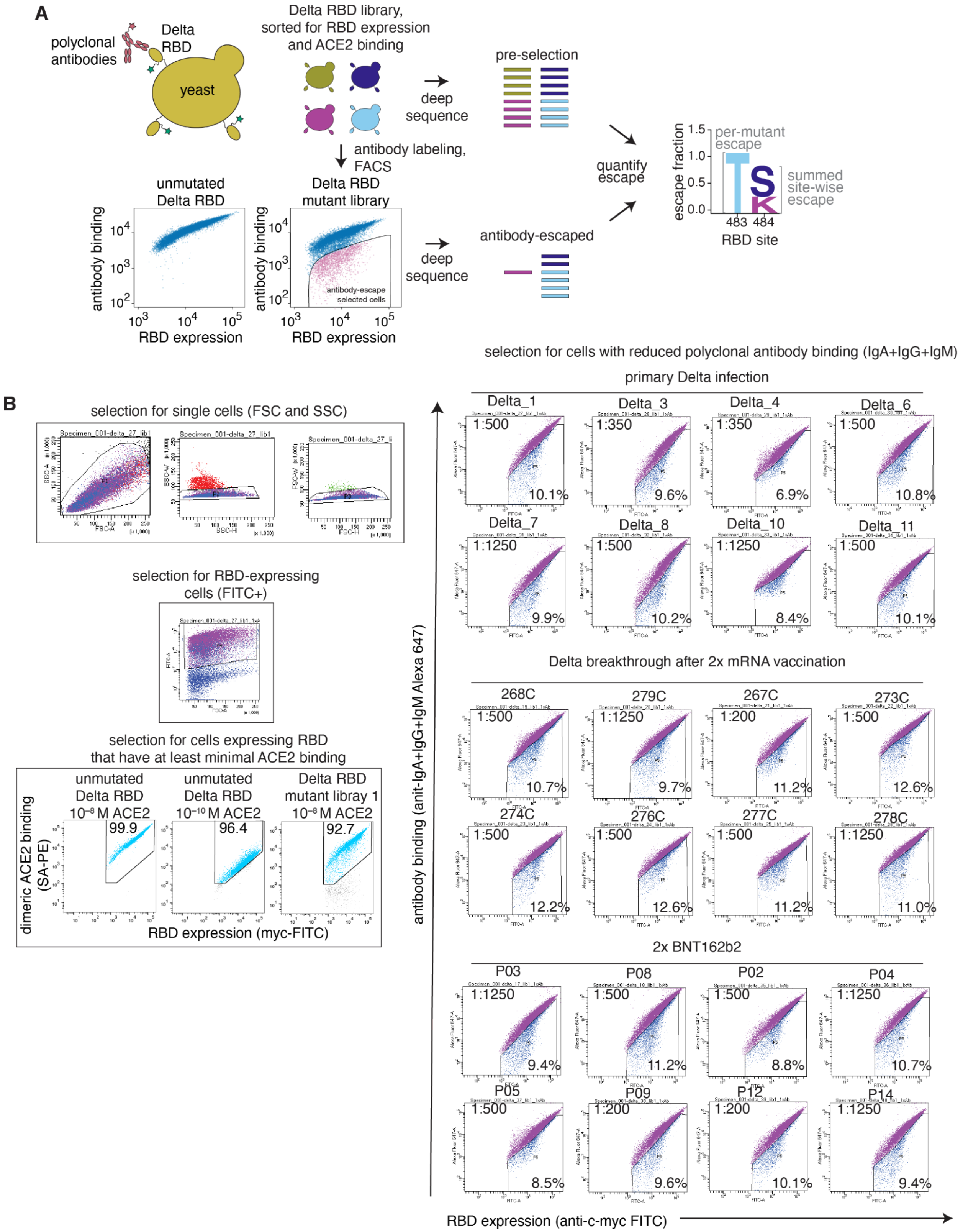

S4 Fig. Deep mutational scanning approach to map mutations that reduce binding of polyclonal plasma antibodies to the Delta RBD. (A) Schematic of the approach. Libraries of

bioRxiv preprint doi: https://doi.org/10.1101/2022.03.12.484088; this version posted March 14, 2022. The copyright holder for this preprint (which was not certified by peer review) is the author/funder, who has granted bioRxiv a license to display the preprint in perpetuity. It is made available under aCC-BY 4.0 International license.

yeast expressing Delta RBD mutants (measured via a C-terminal MYC tag, green star) were incubated with polyclonal antibodies from plasmas or sera and fluorescence-activated cell sorting (FACS) was used to enrich for cells expressing RBD with reduced antibody binding, as detected using an IgA+IgG+IgM secondary antibody. Deep sequencing was used to quantify the frequency of each mutation in the pre-selection and antibody-escape cell populations. The escape fraction represents the fraction of cells expressing RBD with that mutation that fell in the antibody escape FACS bin. Experimental and computational filters were used to remove RBD mutants that were misfolded or unable to bind the ACE2 receptor. **(B)** Top left: Representative plots of nested FACS gating strategy used for all plasma selection experiments to select for RBD-expressing single cells. Samples were gated by SSC-A versus FSC-A, SSC-W versus SSC-H, and FSC-W versus FSC-H) that also express RBD (FITC-A vs. FSC-A). Bottom left: The RBD mutant libraries were sorted to retain cells expressing variants that bound to ACE2 with at least nominal affinity. The RBD mutant libraries were incubated with dimeric ACE2 at 10^{-8} M and FACS-enriched for cells that bound ACE2 with at least as much fluorescence intensity as cells expressing unmutated Delta RBD that were incubated with 10^{-10} M ACE2, to purge the mutant libraries of highly deleterious mutations (i.e., those that have <1% the affinity of unmutated Delta RBD). This retained ~93% of RBD-expressing fractions of the libraries. Right: FACS gating strategy for one of two independent libraries to select cells expressing RBD mutants with reduced binding by polyclonal sera (cells in blue), as measured with an anti-IgA+IgG+IgM secondary antibody. Gates were set manually during sorting. Selection gates were set to capture cells with reduced antibody binding for their degree of RBD expression. FACS scatter plots were qualitatively similar between the two libraries. Serum dilutions used in selections are indicated. SSC-A, side scatter-area; FSC-A, forward scatter-area; SSC-W, side scatter-width; SSC-H, side scatter-height; FSC-W, forward scatter-width; FSC-H, forward scatter height; FITC-A, fluorescein isothiocyanate-area.

bioRxiv preprint doi: https://doi.org/10.1101/2022.03.12.484088; this version posted March 14, 2022. The copyright holder for this preprint (which was not certified by peer review) is the author/funder, who has granted bioRxiv a license to display the preprint in perpetuity. It is made available under aCC-BY 4.0 International license.

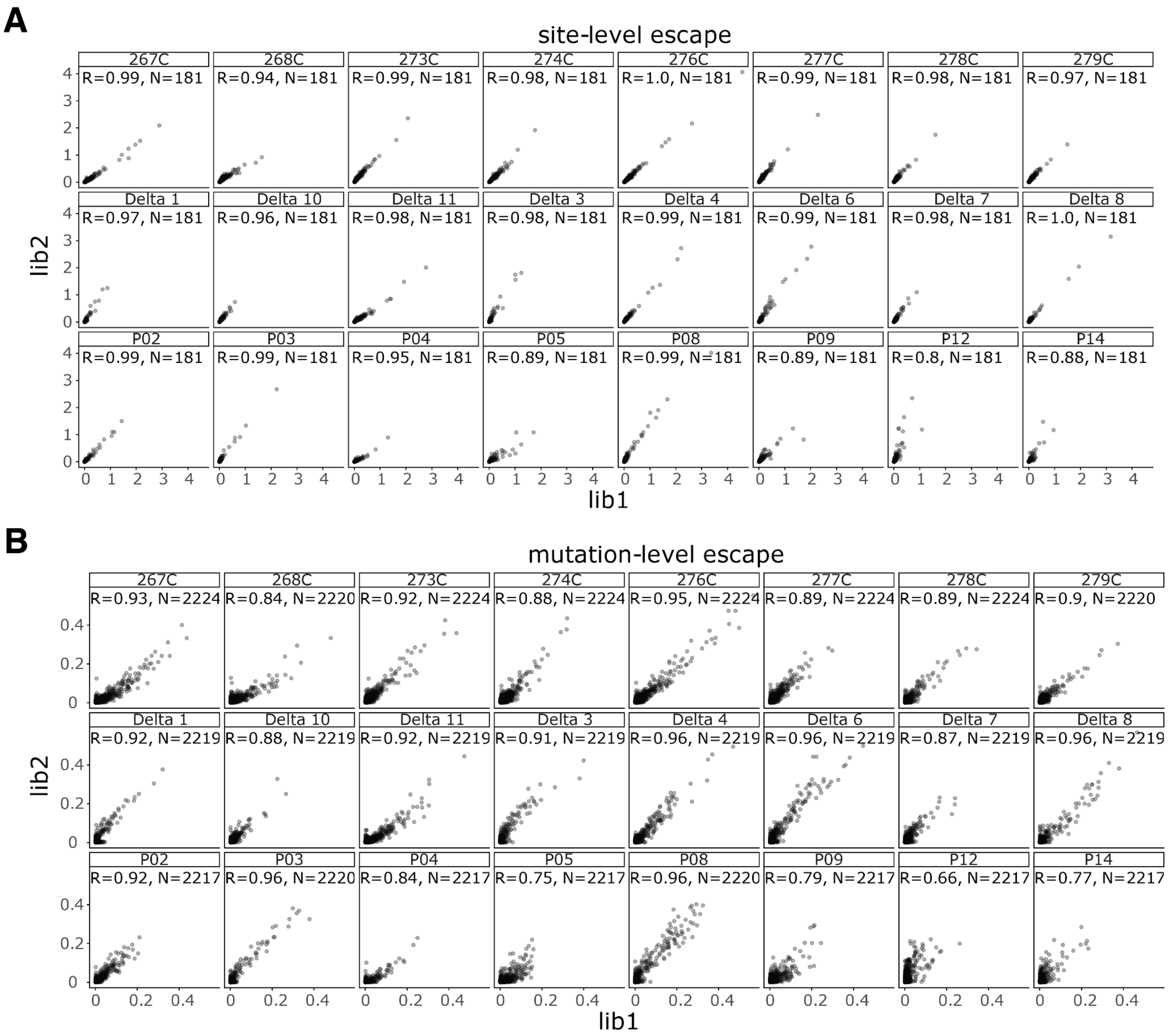

S5 Fig. Correlations of antibody-escape scores between replicates. (A) Site- and **(B)** mutation-level correlations of escape scores between two independent biological replicate libraries. The complete antibody-escape scores are in **S3 Data** and online at https://github.com/jbloomlab/SARS-CoV-2-RBD_Delta/blob/main/results/supp_data/aggregate_raw_data.csv.

bioRxiv preprint doi: https://doi.org/10.1101/2022.03.12.484088; this version posted March 14, 2022. The copyright holder for this preprint (which was not certified by peer review) is the author/funder, who has granted bioRxiv a license to display the preprint in perpetuity. It is made available under aCC-BY 4.0 International license.

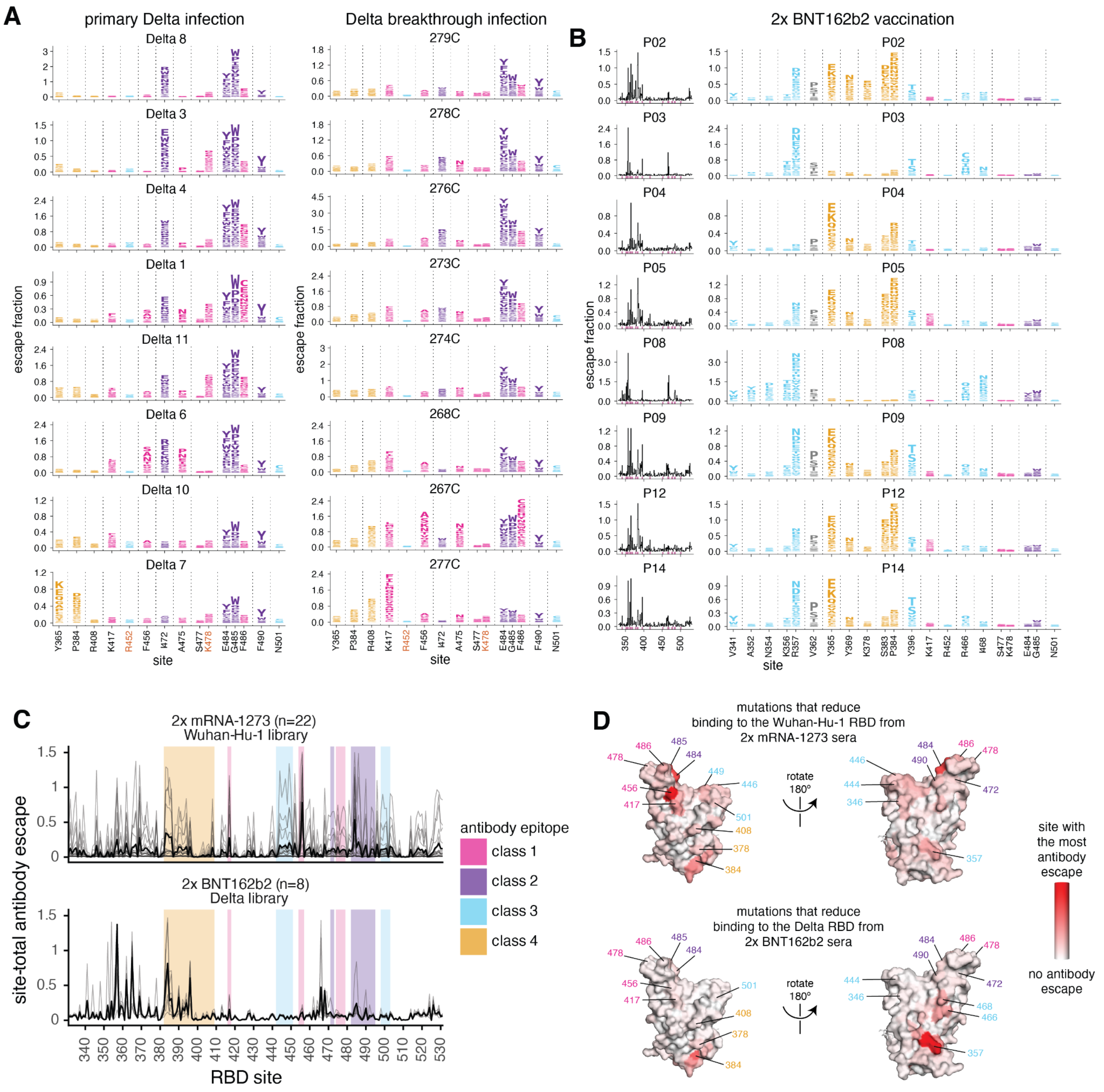

S6 Fig. Complete maps of mutations that reduce binding of polyclonal serum antibodies to the Delta RBD. Delta mutant library escape maps for sera from individuals with **(A)** primary or breakthrough Delta infections after 2x mRNA vaccination (replicated here from **Fig 3 and Fig 5** to facilitate direct comparison) or **(B)** 2x BNT162b2 vaccination. Sites of strong antibody escape (see Methods) for any of the 8 plasmas in A or B are highlighted with pink in the line plots at left and shown in the logo plots at right. Sites 417, 452, 484, 477, 478, and 501 are included in the logo plots whether or not they are sites of strong escape due to their high frequency in circulating viral isolates. **(C)** The average site-total antibody escape for plasmas from individuals vaccinated with 2x mRNA-1273 against the Wuhan-Hu-1 mutant libraries (previously published in (29)) or 2x BNT162b2 against the Delta mutant libraries. Key epitopes

bioRxiv preprint doi: https://doi.org/10.1101/2022.03.12.484088; this version posted March 14, 2022. The copyright holder for this preprint (which was not certified by peer review) is the author/funder, who has granted bioRxiv a license to display the preprint in perpetuity. It is made available under aCC-BY 4.0 International license.

are shaded. **(D)** The average site-total antibody escape mapped to the surface of the Wuhan-Hu-1 RBD (PDB 6M0J, (64)), with red indicating the site with the strongest antibody escape, and white indicating no escape. Key sites are labeled, with labels colored according to antibody epitope. The vaccine sera in B–D are from individuals who were not exposed to the Delta spike via infection or vaccination. The L452R mutation in the Delta RBD can disrupt antibody binding to both the class 2 and class 3 antibody epitopes (24,42), and thus the 2x BNT162b2 (x Delta mutant libraries) plasmas are mostly escaped by mutations in the class 4 epitope (including sites 365, 383, 384) or a non-canonical class 3 epitope that includes site 357. The antibody-escape maps against the Delta RBD mutant libraries are newly generated in this study, whereas the 2x mRNA-1273 antibody-escape maps against the Wuhan-Hu-1 RBD mutant libraries were first reported in (29) and are reanalyzed here.

bioRxiv preprint doi: https://doi.org/10.1101/2022.03.12.484088; this version posted March 14, 2022. The copyright holder for this preprint (which was not certified by peer review) is the author/funder, who has granted bioRxiv a license to display the preprint in perpetuity. It is made available under aCC-BY 4.0 International license.

A

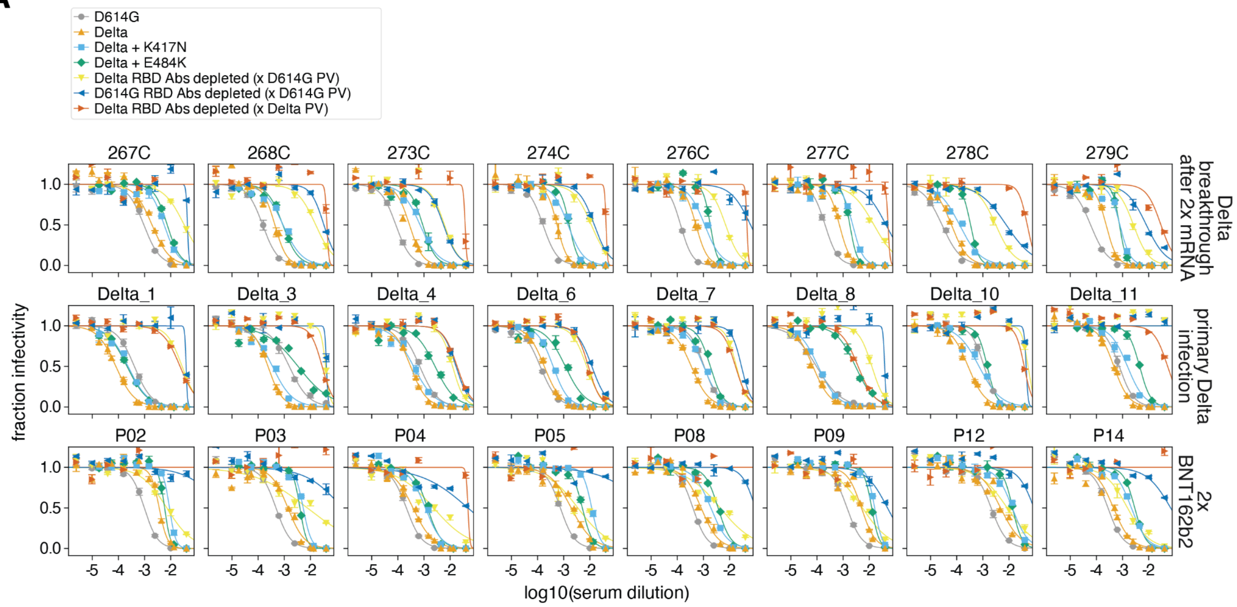

B

spike background	exposure history	fold -change NT50	
		K417N	E484K
Delta	2x BNT162b2	3.5	3.2
	Delta breakthrough after 2x mRNA	2.6	3.3
	primary Delta infection	2.2	8.4
D614G	2x mRNA-1273 (day 30–60)	0.76	2.8
	ancestral infection (day 30–60)	0.84	8.7
	ancestral infection (day 100–150)	1.09	16.1

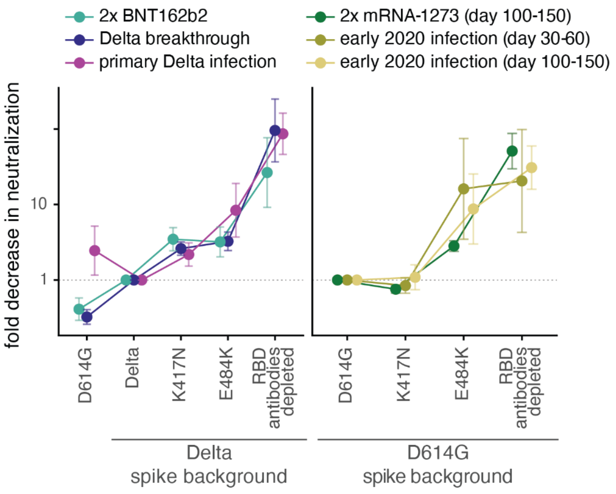

S7 Fig. Neutralization of plasmas against spike-pseudotyped lentiviral particles. (A) Raw neutralization curves for assays shown in **Figure 2 and 6 and S2. (B)** Left: Table summarizing the effect of the K417N and E484K mutations in the Delta and D614G spike backgrounds on neutralization. Right: summary plot of the geometric mean across cohorts of the fold-decrease in neutralization compared to Delta spike (left axis) or D614G spike (right axis). Error bars represent geometric standard deviation. The neutralization assays with 2x BNT162b2, Delta breakthrough, and Delta primary infection were newly performed in this study, and assays with 2x mRNA-1273 and early 2020 plasmas were performed in (12,29) and are reanalyzed here. Neutralization titers are in **S1 Data** and online at https://github.com/jbloomlab/SARS-CoV-2-RBD_Delta/blob/main/experimental_data/results/neut_titers/combined_neut_titers.csv.

bioRxiv preprint doi: https://doi.org/10.1101/2022.03.12.484088; this version posted March 14, 2022. The copyright holder for this preprint (which was not certified by peer review) is the author/funder, who has granted bioRxiv a license to display the preprint in perpetuity. It is made available under aCC-BY 4.0 International license.

Supplementary Files

S1 Data. Neutralization titers for all neutralization assays performed or analyzed in this study. This includes assays newly performed in this study on sera or plasmas from individuals with 2x BNT162b2 vaccination, Delta breakthrough after 2x mRNA vaccination, or Delta primary infections before and after depletion of D614G (Wuhan-Hu-1) or Delta RBD-binding antibodies or against point mutants in the Delta spike background. This table also includes previously published neutralization titers from individuals infected with early 2020 viruses (23), vaccinated 2x with mRNA-1273 (29), and individuals with a primary Beta infection (12). This table is also available at:
https://github.com/jbloomlab/SARS-CoV-2-RBD_Delta/blob/main/experimental_data/results/neut_titers/combined_neut_titers.csv

S2 Data. The effects of all single amino-acid mutations in the Delta RBD on ACE2 binding and RBD expression. These results are also published in (36). This file is also available at:
https://github.com/jbloomlab/SARS-CoV-2-RBD_Delta/blob/main/results/final_variant_scores/final_variant_scores.csv.

S3 Data. Plasma-escape scores for sera or antibodies mapped against the Wuhan-Hu-1, Beta, or Delta RBD single-mutant libraries. The serum-escape scores for sera/plasmas mapped against the Delta RBD libraries are new to this study. The data for antibodies or sera/plasmas mapped against the Wuhan-Hu-1 or Beta RBD libraries were previously published (12,22–24,29,42–44). This table is also available at
https://github.com/jbloomlab/SARS-CoV-2-RBD_Delta/blob/main/results/supp_data/aggregate_raw_data.csv
